# Analysis of the stoichiometric metal activation of methionine aminopeptidase

**DOI:** 10.1186/1471-2091-10-32

**Published:** 2009-12-17

**Authors:** Sergio C Chai, Qi-Zhuang Ye

**Affiliations:** 1 Department of Biochemistry and Molecular Biology, Indiana University School of Medicine, Indianapolis, Indiana 46202, USA

## Abstract

**Background:**

Methionine aminopeptidase (MetAP) is a ubiquitous enzyme required for cell survival and an attractive target for antibacterial and anticancer drug development. The number of a divalent metal required for catalysis is under intense debate. *E. coli *MetAP was shown to be fully active with one equivalent of metal by graphical analysis, but it was inferred to require at least two metals by a Hill equation model. Herein, we report a mathematical model and detailed analysis of the stoichiometric activation of MetAP by metal cofactors.

**Results:**

Because of diverging results with significant implications in drug discovery, the experimental titration curve for Co^2+ ^activating MetAP was analyzed by fitting with a multiple independent binding sites (MIBS) model, and the quality of the fitting was compared to that of the Hill equation. The fitting by the MIBS model was clearly superior and indicated that complete activity is observed at a one metal to one protein ratio. The shape of the titration curve was also examined for activation of metalloenzymes in general by one or two metals.

**Conclusions:**

Considering different scenarios of MetAP activation by one or two metal ions, it is concluded that *E. coli *MetAP is fully active as a monometalated enzyme. Our approach can be of value in proper determination of the number of cations needed for catalysis by metalloenzymes.

## Background

A myriad of enzymes exploit the diverse and dynamic properties of metal ions for activity, many of which carry out crucial functions for cellular survival [1]. An important feature in the understanding of their catalytic mechanisms entails the number of a metal ion that the active site needs for complete activation. A challenge transpires when the cofactor is loosely bound, making it difficult to perform direct metal speciation on purified enzyme. In the case of metalloenzymes, the number of an activating metal cofactor can be deduced from stoichiometric titration curves, where the rise in activity correlates with increasing metal concentrations [2]. Herein, we report the application of a robust nonlinear regression approach in determination of the number of the catalytically relevant metal ion required by the metalloprotease methionine aminopeptidase (MetAP).

MetAP is involved in protein maturation by catalyzing the hydrolytic excision of the N-terminal methionine from nascent proteins [3], which was demonstrated to be an essential process [4-6]. The MetAP apoenzyme can be activated by a number of divalent cations, including Co^2+^, Mn^2+^, Fe^2+^, Ni^2+ ^and Zn^2+ ^[7,8]. The majority of MetAP inhibitors discovered in the quest to develop antibacterial and anticancer agents bind to the active site, interacting directly with the catalytic metal cofactor. However, most of the compounds that inhibit a purified enzyme cannot show their effect at the cellular level [9]. This was inferred to be partly due to discrepancies in the type and number of the cation used during *in vitro *assays and those found under physiological conditions [9,10]. Although most of MetAP structures showed an active site containing two metal ions [11], *E. coli *MetAP was proposed to contain only one metal based on measurement of activities in solution as a function of metal concentrations [12]. However, it was suggested to be inconclusive due to the small number of data points [13]. Because metal stoichiometry is more accurately determined under a tight-binding situation, we previously determined a one metal per enzyme ratio by graphical analysis of a titration curve under the tight-binding condition [14]. The result was disputed because computation of the Hill coefficient based on the same titration curve indicated that this enzyme required at least two metal ions [13]. As a result of the diverging outcomes with significant implications in MetAP inhibitor development, the correct assignment of the number of the activating metal ion in MetAP becomes very significant. Therefore, we provide here a detailed analysis of the stoichiometric activation of *E. coli *MetAP by binding of Co^2+^. Our conclusion is that *E. coli *MetAP requires only one equivalent of metal ions for full activation.

## Results and discussion

### Scenarios in the activation of a metalloenzyme in an apoform by one or two metals

Stoichiometric titration curves have been widely employed in the determination of the number of a metal ion required for activation of metalloenzymes [2]. A general procedure involves generation of a curve in terms of enzymatic activity as a function of the metal concentrations added. Because the protein concentration is known, the x-axis can be expressed in terms of metal/protein ratios. It is important to note that for accurate stoichiometric titrations, the enzyme concentration should be at least ten times higher than the equilibrium dissociation constant, to approach the tight-binding condition [2]. We recently described a nonlinear curve fitting of metal titration curves using the multiple independent binding sites (MIBS) model to determine the equilibrium binding affinity, as a *K*_D _value, based on functional enzyme concentrations [15].

**Figure 1:**
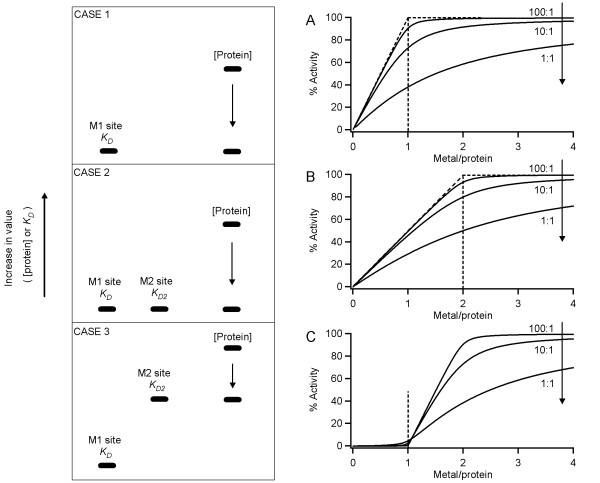
**Three different scenarios in metal activation of a metalloenzyme**. The enzyme is activated by only a single metal per protein (Case 1) or by two metals (Cases 2 and 3). In each of the three cases, vertical positions represent the relative values of protein concentration, and *K*_D _and *K*_D2 _for the first and second metal sites M1 and M2. (**A**) Simulated stoichiometric titrations for Case 1. The *K*_D _value was set to 0.2 μM with *n *= 1, and the protein concentration was set at 20, 2 or 0.2 μM (yielding 100:1, 10:1 or 1:1 [protein]/*K*_D _ratio). (**B**) Simulated stoichiometric titrations for Case 2. Both the *K*_D _and *K*_D2 _values were fixed at 0.2 μM, with the total protein concentration of 20, 2 or 0.2 μM (resulting in 100:1, 10:1 or 1:1 [protein]/*K*_D _ratio) and *n *= 2. **(C) **Simulated stoichiometric titrations for Case 3. The *K*_D _value for the tight metal site M1 was fixed at 0.2 μM, and the *K*_D2 _value for the weaker site was set at 20 μM, with the total protein concentration of 2000, 200 or 20 μM, giving a 100:1, 10:1 or 1:1 [protein]/*K*_D2 _ratio.

When considering different situations that take into account enzyme activation by one or two metals, one can envision the following scenarios in Cases 1-3 that affect the shape of a stoichiometric titration curve (Fig. 1). The simplest scenario is Case 1, where the apoenzyme is activated by a single metal ion. When the protein concentration is fixed at more than 10 times higher than the *K*_D _value for the cation to the metal site, it creates a tight binding situation. Under the tight binding condition, a cation that is present at low concentrations binds in a stoichiometric fashion to the enzyme, resulting in the observed linear onset of the curve (Fig. 1A, upper-most curve). Data extrapolation of this linear onset intercepts the maximal activity (Fig. 1A, diagonal and horizontal dashed lines), and the endpoint for the titration would indicate the metal/protein molar ratio (Fig. 1A, vertical dashed line). Visual interpretation of this type of curves should suffice in order to obtain the stoichiometric information, and this approach has been widely employed and accepted as a robust graphical analysis method [2]. Using Eq. (4), we have simulated the behavior of titration curves at different ratios of the protein concentration to *K*_D _(Fig. 1A). When the protein concentration is close to the binding affinity (ratio of 1:1), the accurate stoichiometric information cannot be extracted from the curves (Fig. 1A, bottom-most curve). However, the accuracy in determination of the number of the activating cation per enzyme progresses as the ratio increases, and a ratio of 100:1 provides an accurate determination of the number of metal required for activity per protein (Fig. 1A, upper-most curve). This trend observed in the curves constructed using Eq. (4) follows the same anticipated pattern as a MIBS model described by Winzor and Sawyer [2].

There are cases when a single metal cannot activate the apoenzyme and two metal ions are required for activation. It is therefore necessary to fill both the first binding site (M1 site) and the second site (M2 site) to observe enzyme activation. Case 2 (Fig. 1) deals with one of the situations when the activation of apoenzyme requires two metal ions with equal or different affinities. The shape of the titration curve will depend on the protein concentration in relation to the cation binding affinities to both metal sites (M1 and M2). When the protein concentration is much higher (10 fold or more) than both of the two dissociation constants (Case 2, Fig. 1B), one would expect a rectangular hyperbola, which clearly indicates a 2:1 metal to protein ratio. This case is exemplified by an aminopeptidase from *Streptomyces griseus*, where the titration curve showed a hyperbolic dependence of activity on metal concentration, and addition of two molar equivalents of metal to apoenzyme fully restored activity [16]. For many dinuclear enzymes, each of the two metals is coordinated by a different set of amino acid residues. Therefore, it is common that a metal ion binds at the two sites with different affinities. However, no matter whether the binding affinities to the M1 and M2 sites are equal or different, a titration curve similar to that obtained for Case 2 (Fig 1B) is achieved as long as the protein concentration is much higher than both *K*_D _and *K*_D2 _values (a tight binding situation).

Case 3 (Fig. 1) represents another situation when binding to the first metal site (M1 site) is much stronger than to the second site (M2 site), due to a wide difference in affinity. A protein concentration can be selected, so that metal binding is tight to the first site but not to the second site. Because filling only the tight metal site does not result in enzyme activation, a lag in the observed activity corresponds to 1 equivalent of metal titrated. The signal arising from the activated enzyme is then as a result of metal binding to the weaker second M2 site. The steepness of this onset depends on the *K*_D2 _value relative to the protein concentration (Fig. 1C, upper vs lower curves). This type of metal-activation mode has been observed in a bacterial hydantoinase that required two metals for activity, while binding of a single cation per monomer failed to activate the enzyme, displaying an activity lag in the titration curve [17].

### Analysis of the stoichiometric titration curve for activation of *E. coli *MetAP by Co^2+^

MetAP belongs to the dinuclear metallohydrolases [1]. Many MetAP structures have been reported with two metal ions at the active site [11], and the two metal ions are coordinated by five conserved amino acid residues (D97, D108, H171, E204 and E235 in *E. coli *MetAP). The affinities of the two metal ions are quite different in solution. Based on electronic absorption spectra titration, isothermal titration calorimetry, and kinetics, D'souza *et al. *showed that dissociation constants for the tighter site were at or below micromolar (*K*_D _0.3 μM, 0.2 μM and 6 μM for Co^2+^, Fe^2+ ^and Mn^2+^, respectively), but the affinity for the second metal ion was much weaker with a millimolar dissociation constant (*K*_D2 _2.5 mM for Co^2+^) [18,19]. During crystallization experiments, a high protein concentration is often required. The high protein concentration facilitates the binding of metal ions to both the first site and the second site. The presence of a substrate or inhibitor can further enhance the affinity of metal ions to the two sites. Therefore, it is not surprising to see the dimetalated MetAP form in crystal structures. By limiting the metal concentration during crystallization, we have obtained a monometalated MetAP structure in complex with an inhibitor [14].

**Figure 2:**
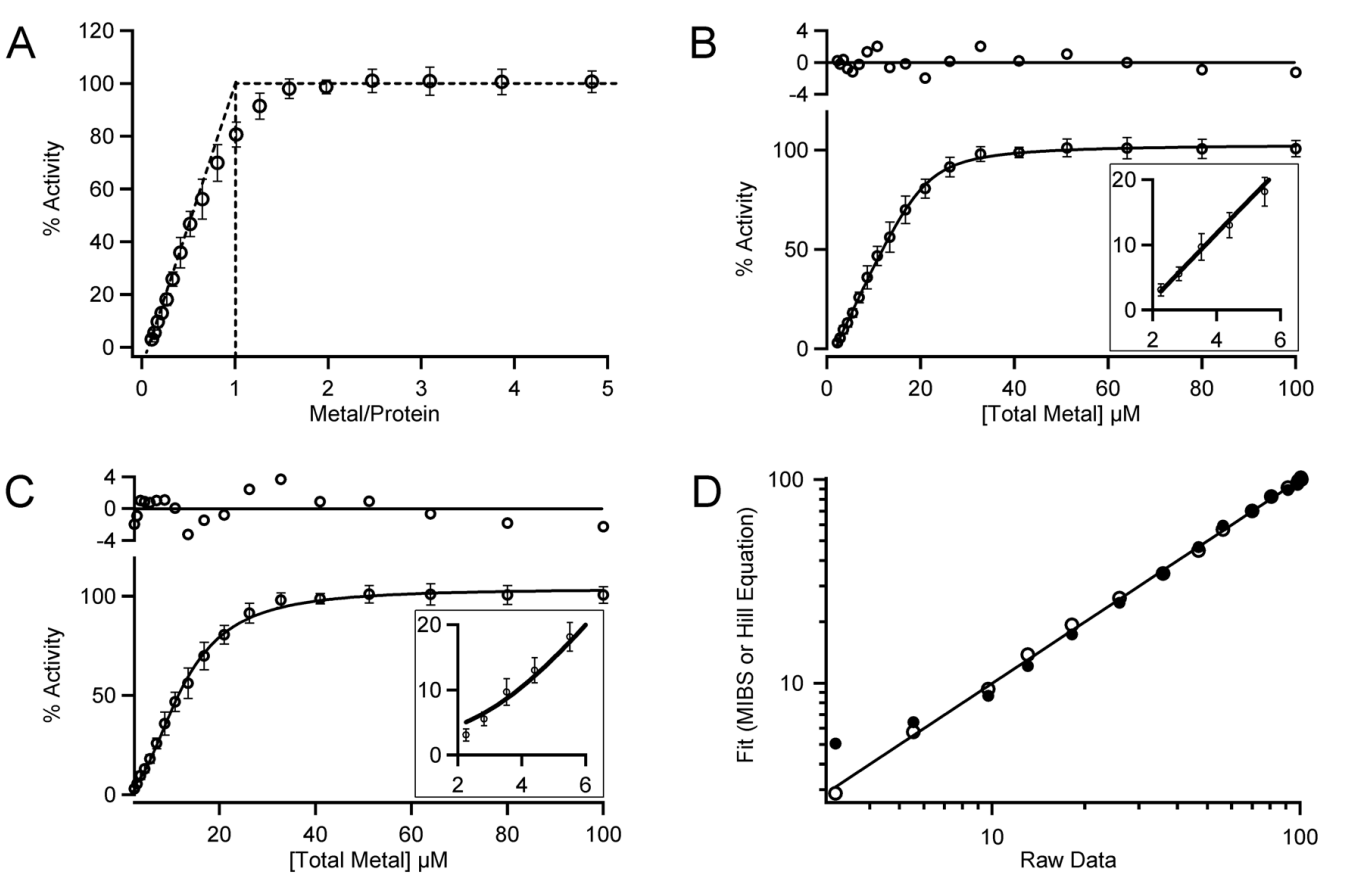
**Curve fitting of the experimental data of activation of *E. coli *MetAP by Co^2+^**. (**A**) *E. coli *MetAP (20 μM) was titrated with increasing concentrations of Co^2+^, and its activity was monitored by fluorescence. This titration curve is adapted from [14]. The linear segment (diagonal dash line) corresponding to data extrapolation intercepts the maximal activity (horizontal dash line). The endpoint for the titration indicates clearly a 1:1 metal/protein molar ratio (vertical dash line). (**B**) The titration data in (A) was fitted with Eq. (4) (the MIBS model), giving *n *= 1.0 and *K*_D _= 0.9 μM. (**C**) The same data was fitted with Eq. (7) (the Hill equation), giving *n *= 2.3 and *K*_0.5 _= 12.1 μM. In both (B) and (C), residuals of the fitting are shown on top of the plots, and fittings at points of low metal concentrations are shown as inserts. (**D**) Computed fit values using both models [values for solid lines in (B) and (C)] were plotted against the raw data in a log-scale format (the MIBS model, open circles; the Hill equation model, filled circles). For a perfect fit, the plot would result in a straight line with a slope value of 1 (solid line), which is obtained by plotting the raw data against itself.

Direct metal speciation on a purified MetAP enzyme is not a viable approach, because of inconsistencies due to metal exchange during the purification process as a result of the relatively weak metal binding affinity. *E. coli *MetAP was proposed to contain only one equivalent of metal based on kinetic measurement of activities as a function of metal concentrations [12]. According to the reported *K*_D _of 0.3 μM for Co^2+^, we used 20 μM of *E. coli *MetAP apoenzyme in the titration (Fig. 2A), which is 67 fold higher than the *K*_D _value. Under such a condition, a cation that was present at low concentrations bound in a stoichiometric fashion to the enzyme, resulting in the observed linear onset of the curve. Fig. 2A is an adaptation of the titration curve reported by us previously [14], and graphical analysis clearly indicates a 1:1 metal/protein molar ratio.

In order to clarify the differing conclusions arising from the same titration curve (graphical analysis [14] as opposed to analysis by the Hill equation [13]), Eq. (4) was used as a robust tool that can be employed at any enzyme concentration. The same titration curve was expressed in terms of the total metal concentration before fitting with Eq. (4) (MIBS model, Fig. 2B) or Eq. (7) (Hill equation, Fig. 2C). The fit with the MIBS model gave *n *= 1.0, indicating one metal per active site, while the calculated Hill coefficient of 2.3 would indicate a minimum of two metals per active site. Hu and coworkers fitted the Hill function to the total metal concentration against the corresponding activity in order to get initial parameters needed to obtain a plot of activity as a function of the free metal concentration [13]. However, the determined initial estimations would have significant effects in the shape of the curve at latter stages of analysis, which possibly led to a different conclusion. They reported the fitting with an initial Hill coefficient of 2.1, and iteration of the data yielded *n *values significantly greater than 2 [13].

Comparison of the residuals obtained using both models (Figs. 2B and 2C inserts) reveals that the Hill equation produced poor data fitting at low metal concentrations, indicating systematic deviation of the fit from the data. The effect becomes more evident when the fits obtained from both models are plotted against the original data in a log-scale (Fig. 2D). In contrast to the reasonable fit by the MIBS model, the Hill equation model showed markedly departure from linearity at low values. We find this type of plot to be very informative as a way to visually describe how well a model fits the data. The Durbin-Watson statistic was employed to assess correlation among the residuals, giving the MIBS model a value of 1.5. On the other hand, the Hill function showed the Durbin-Watson statistic of 0.8. Further departure from the ideal value of 2 by the Hill function indicates higher correlation among residuals. Another method widely used to check for randomness of residuals is the Wald-Wolfowitz runs test [20-22]. Out of 18 residual points, there are 8 runs obtained from modeling with MIBS (*P *= 0.251) as opposed to only 5 runs for that modeled by the Hill equation (*P *= 0.013). The latter model was associated with a *P *value < 0.05, confirming systematic deviation.

It is evident from the analysis presented in Fig. 2 that our proposed MIBS model correlates with the results obtained by graphical analysis from the titration curve of activities as a function of metal/protein ratios (Fig. 2A). Certainly, the curve based on the raw data (Fig. 2A) has the same profile as the curves presented in Cases 1 and 2 (Figs. 1A and 1B), and it does not follow the behavior associated to Case 3 (Fig. 1C). The reported binding affinity of Co^2+ ^to the second binding site M2 (*K*_D _of 2.5 mM) of *E. coli *MetAP is over 8,000 times weaker than that for the first metal site M1 (*K*_D _of 0.3 μM). Taking into consideration the total enzyme concentration used in the generation of Fig. 2A (20 μM), a tight-binding situation was created for the M1 site, but not for the M2 site. If two metal ions are required for MetAP activation, the shape of the curve would therefore resemble that for Case 3 (Fig. 1C). The dissociation constant reported for Co^2+ ^to the second weaker site (vide supra) was obtained spectroscopically in the absence of a substrate. However, in the event that the presence of substrate decreased the *K*_D _value for the second binding site to such extent that a tight binding situation was created for both metal sites, then our proposed MIBS model would determine *n *= 2 and the raw data (Fig. 2A) would bear a resemblance to Fig. 1B.

Several factors can potentially affect the shape of a titration curve, such as protein aggregation and residual metal or metal chelator in an apoprotein preparation, and lead to different conclusions according to fitting of the curves. *E. coli *MetAP is a very soluble protein, and it was used as high as 1 mM in equilibrium dialysis without interference of aggregation [23]. In the equilibrium dialysis study, it was concluded that the enzyme bound up to 1.1 equivalent of Co^2+ ^in the metal concentration range likely to be found in vivo, which is consistent with our conclusion of 1:1 stoichiometry. We recently characterized MetAP from *Mycobacterium tuberculosis *for metal binding and activation and found that it is also a monometalated enzyme [15].

## Conclusions

Metal cofactors form integral parts in a great variety of enzymes, and the correct assignment of the number of an activating metal is an essential progression towards understanding mechanistic aspects, and in the case of MetAP, towards the development of lead candidates for drug discovery. We have applied the MIBS model with a more cautious interpretation of the fits, arriving at the conclusion that a single metal ion per active site is sufficient to restore the complete *E. coli *MetAP activity. We have also outlined the interplay among *K*_D _values and the total protein concentration in defining the shape of the titration curve, which is illustrated by different scenarios involving one- or two-metal activation. We believe that these assessments are relevant in the proper analysis of stoichiometric binding and activation of metalloenzymes by metal ions.

## Methods

MetAP apoenzyme can be instantly activated by a metal ion, such as Co^2+^, and the activity can be monitored by fluorescence (λ_ex_/λ_em_, 360/460 nm) using a fluorogenic substrate methionyl aminomethylcoumarin (Met-AMC) [8]. Fluorescence signal arising from the substrate hydrolysis with increasing concentrations of metal produces a rectangular hyperbola (Fig. 2A), which can be analyzed using the MIBS binding equation as Eq. (1) [24].(1)

where *K*_D _is the dissociation constant, *n *is the number of independent binding sites, [*MP*] is the concentration of bound metal, [*M*]_F _and [*P*]_T _are the concentrations for free metal and total protein, respectively.

Eq. (1) can be expressed in terms of the total metal concentration, giving Eq. (2), which was successfully applied to calculate the number of receptors per cell in titrations analyzed by fluorescence activated cell sorting [24].(2)

Fluorescence signal *F *resulting from the substrate hydrolysis can be adapted in Eq. (3) as the fraction of sites occupied per total number of the binding site, *θ*, taking into account the signal from a blank *F*_0 _and the maximal signal *F*_MAX_. *F *can be calculated by Eq. (4).(3)

Eq. (4) was used to fit the raw data points that make up the *E. coli *MetAP titration curve (Fig. 2B). This equation was also utilized to generate simulated curves for Cases 1 and 2 (Figs. 1A-B). Eq. (4) is applicable to assay conditions at any given protein concentration, and it allows for simplification in curve fitting, because concentrations are based on the *total *protein and metal in the assay solution instead of the *free *(unbound) species. Therefore, the need for conversion from the total concentration to the free concentration of individual species is avoided.

In case the second metal ion is required for activation and has a much weaker affinity, Eq. (4) was modified as Eq. (5).(5)

where *K*_D2 _is the dissociation constant of the second metal and [*M*]_R _is the remaining free metal concentration after metal binding to the first metal site, as calculated by Eq. (6). Eq. (5) was used to obtain simulated curves that describe Case 3 (Fig. 1C).(6)

The metal titration data (Fig. 2C) was fitted to Eq. (7) (the Hill equation), where *K*_0.5 _is [*M*]_T _at *F*_MAX_/2.(7)

Curve fitting and analysis were performed using Igor Pro (Wavemetrics, Lake Owego, OR) and Sigma Plot (Systat Software, San Jose, CA). Simulated curves were generated by Microsoft Excel.

## Authors' contributions

SCC was involved in writing the manuscript, the design and curve fit analysis of the mathematical models for stoichiometric metal titrations. QZY supervised the project and wrote the manuscript. All authors read and approved the final manuscript.
